# Sex and Intersectional Disparities in Access to Deceased Donor Kidney Transplantation: A 17‐Year Population‐Based Cohort Study

**DOI:** 10.1111/nep.70276

**Published:** 2026-09-03

**Authors:** Tennille L. Vitagliano, Nicole L. De La Mata, Peter S. Hsu, Stephen I. Alexander, Kate Wyburn, Melanie L. R. Wyld

**Affiliations:** ^1^ Faculty of Medicine and Health The University of Sydney Sydney New South Wales Australia; ^2^ Department of Allergy and Immunology and the Kids Research Institute The Children's Hospital at Westmead Westmead New South Wales Australia; ^3^ Centre for Kidney Research, Kids Research Institute and the Department of Nephrology The Children's Hospital at Westmead Westmead New South Wales Australia; ^4^ Department of Renal Medicine Royal Prince Alfred Hospital Sydney New South Wales Australia

**Keywords:** deceased donor, intersectionality, kidney transplant, sex inequality, waitlisting

## Abstract

**Background:**

While sex disparities in access to transplant have been shown in a number of countries, the ways additional measures of disadvantage, such as intersectional and clinical disadvantage, influence sex‐based disparities is less well understood. Access to transplant by sex or intersectional disadvantage has not been studied in Australia.

**Methods:**

We conducted a population‐based cohort study of incident dialysis patients (2006–2023) using Australia & New Zealand Dialysis and Transplant Registry data. Cox proportional hazards models, interaction analyses and competing‐risk approaches were used to evaluate associations between sex, waitlisting and deceased donor transplantation, adjusting for intersectional and clinical characteristics.

**Results:**

Among 47 884 incident dialysis patients contributing 147 510 person‐years of follow‐up, females were 19% less likely to be waitlisted than males (aHR 0.81, 95% CI 0.77–0.84). Sex‐based disparities were greatest among females with intersectional disadvantage, including ethnic minority status (Aboriginal & Torres Strait Islander: aHR 0.57, 95% CI 0.49–0.66), diabetic kidney disease (aHR 0.61, 95% CI 0.56–0.67), having ≥ 3 comorbidities (aHR 0.66, 95% CI 0.56–0.79) and obesity (aHR 0.69, 95% CI 0.65–0.75), compared to male peers. Once waitlisted, females and males had similar likelihood of deceased donor transplantation (aHR 1.04, 95% CI 0.99–1.10) and comparable waitlist mortality (aHR 0.91, 95% CI 0.76–1.08).

**Conclusions:**

Females in Australia experience substantially reduced access to the kidney transplant waitlist, with the greatest inequities affecting those facing overlapping social and/or clinical disadvantage. The absence of sex‐disparity after waitlisting indicates that inequities arise earlier in the referral and evaluation pathway. Interventions to improve equity must target these upstream stages and address intersecting drivers of disadvantage.

## Summary at a Glance

- Female dialysis patients in Australia were less likely than males to be activated on the kidney transplant waitlist.
- Sex disparities were greatest among those with intersecting social and clinical disadvantage.
- Similar transplant rates after activation suggest inequities arise prior to waitlisting, supporting the need for targeted upstream interventions.

**Abbreviations**
aHR: adjusted hazard ratio
ANZDATA: Australia & New Zealand Dialysis and Transplant Registry
BMI: body mass index
CAD: coronary artery disease
CI: confidence Intervals
cPRA: calculated panel reactive antibody
CVD: cerebrovascular disease
GN: glomerulonephritis
HR: hazard ratio
IQR: interquartile range
PVD: peripheral vascular disease

## 1 Introduction

Kidney transplantation is the optimal treatment for people with kidney failure, offering survival benefits, improved quality of life and increased life participation [1]. However, deceased donor kidneys remain a scarce resource and their equitable allocation remains a central challenge for transplant systems worldwide [2].

Sex is among the most consistently demonstrated axes of inequity in this allocation, with international evidence demonstrating lower access to transplantation for females across diverse settings [3, 4, 5, 6, 7, 8, 9]. Yet these disparities are unlikely to be uniform. Intersectionality, a framework originating in Black feminist scholarship, holds that inequities arise through intersecting structures of power, rather than from social identities experienced in isolation [10], such that sex, ethnicity, socio‐economic position and related categories combine to produce cumulative disadvantage [11]. This insight has propelled the growing application of intersectional approaches in health disparities research [11] and it implies that average estimates of sex‐based disadvantage may conceal substantially greater inequity among females who face additional forms of marginalisation.

A complete account of access to transplant must also extend this lens to clinical characteristics. Factors such as comorbidity burden, cause of kidney failure and obesity are not themselves forms of structural disadvantage, but they constitute part of the criteria for which transplant eligibility is assessed and they are unevenly distributed across social groups. Where clinical thresholds are applied differently by sex, or where clinical disadvantage clusters with social disadvantage, these factors may amplify, rather than merely explain, sex‐based inequity. Examining intersectional and clinical dimensions together is therefore necessary to identify which females are the most disadvantaged and at what point in the transplant pathway—evidence needed to inform policies and models of care directed at those facing the greatest barriers.

Australia is well placed to address these questions due to the near‐complete capture of dialysis, waitlisted and transplanted patients through the Australian and New Zealand Dialysis and Transplant (ANZDATA) registry [12]. Despite this, contemporary national data examining sex‐based disparities in access to deceased donor transplantation are limited and no study, in Australia or internationally, has systematically examined whether sex‐based disadvantage is magnified at the intersection of social and clinical characteristics.

This study examines sex‐based disparities in access to deceased donor kidney transplantation in Australia. We explored the association between sex and (i) activation on the kidney transplant waitlist, (ii) time from dialysis initiation to waitlisting and (iii) receipt of a deceased donor transplant once waitlisted. We hypothesised that the association between sex and access to transplantation would vary across social and clinical groups, with the greatest disadvantage experienced by females facing intersecting social and/or clinical disadvantage.

## 2 Materials and Methods

### 2.1 Study Population & Design

**FIGURE 1:**
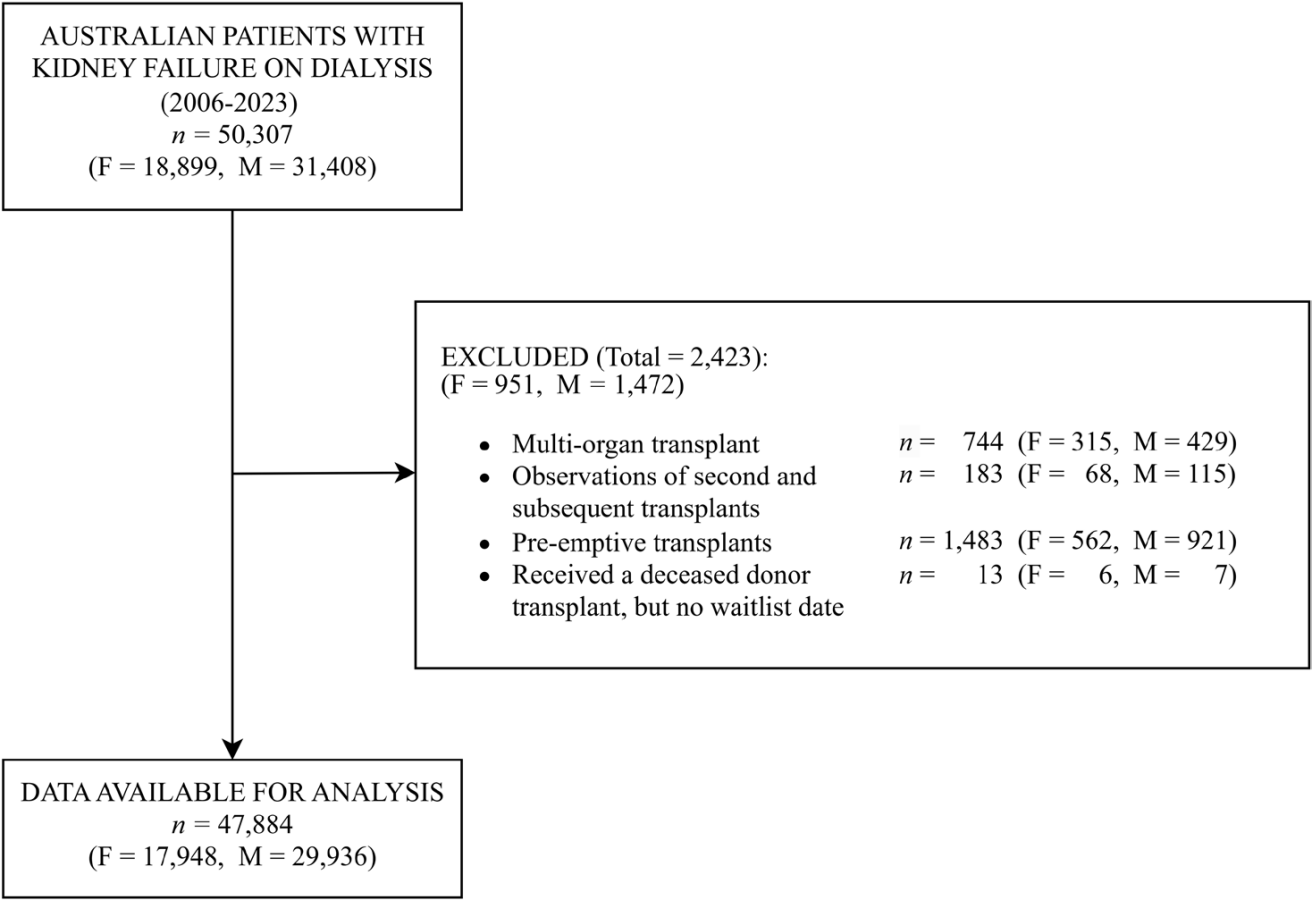
Flow diagram of study selection.

We conducted a population‐based cohort study of incident dialysis patients in Australia who commenced between 28 June 2006 and 31 December 2023. We excluded people who received a multi‐organ transplant, a pre‐emptive transplant, had data pertaining to a second or subsequent graft or received a deceased donor transplant without a recorded date of waitlisting (Figure 1). Ethics approval was obtained from the Western Sydney Local Health District Human Research Ethics Committee (PID01571‐2022/ETH01396). Reporting followed STROBE guidelines [13].

### 2.2 Data Collection

Data were obtained from ANZDATA, a national clinical registry of patients with kidney failure in Australia and New Zealand, with previously described data collection and validation methods [14]. Sex/gender was obtained from ANZDATA, where the variable is recorded as ‘gender’ (formerly ‘sex’) and classified as male, female, or intersex. As the registry does not distinguish sex assigned at birth from gender identity and the variable is clinician‐entered, we treated this measure as sex in analyses while acknowledging the conceptual distinction between sex and gender.

### 2.3 Outcomes

Primary outcomes were: (i) activation on the kidney transplant waitlist; (ii) time from dialysis initiation to waitlisting and (iii) receipt of a deceased donor transplantation after waitlisting. Secondary outcomes included: (iv) time to transplantation once waitlisted and (v) death while on the waitlist.

### 2.4 Covariates and Conceptual Framework

Covariates were selected a priori based on theoretical relevance to transplant access: age group (< 18, 18–45, 46–65, > 65 years), ethnicity (Australian & New Zealander/European, Aboriginal & Torres Strait Islander, Māori & Pacific Peoples, Asian and Other), socio‐economic position (most disadvantaged, least disadvantaged), medical comorbidities (diabetes, cerebrovascular disease (CVD), peripheral vascular disease (PVD), coronary artery disease (CAD), chronic lung disease, cancer pre‐dialysis), comorbidity burden (0–3 or more), cause of kidney failure (diabetic kidney disease, hypertension/renal vascular disease, glomerulonephritis (GN), familial/hereditary kidney diseases, tubulointerstitial diseases and Other), BMI group (‘non‐obese < 30 kg/m^2^’ or ‘obese ≥ 30 kg/m^2^’), smoking status (current, former, never), late referral (< 3 months before dialysis), dialysis modality (peritoneal, haemodialysis) and calculated panel reactive antibody (cPRA; 0–19, 20–84, 85–94, 95–98, ≥ 99).

Comorbidity burden (0–3 or more) represented the total number of listed comorbidities, excluding type‐2 diabetes to avoid collinearity with diabetic kidney disease. Socio‐economic position was generated from SEIFA: Index of Relative Socio‐economic Disadvantage deciles and dichotomised into ‘lowest 20% (most disadvantaged)’ and ‘highest 80% (least disadvantaged)’.

Effect modification was assessed across two domains: intersectional (ethnicity, socio‐economic position) and clinical (age, cause of kidney failure, comorbidity burden, BMI). We evaluated whether associations between sex and (i) waitlisting and (ii) deceased donor transplantation varied across these factors.

### 2.5 Statistical Analyses

Descriptive statistics were reported as frequencies and proportions for categorical variables, stratified by sex, for both dialysis and waitlisted cohorts. Median time to each outcome [interquartile range, IQR] was estimated using Kaplan–Meier survival functions, with sex differences assessed using log‐rank tests.

Cox proportional hazards models were used to evaluate the association between sex and each primary outcome. Two time‐to‐event models were constructed: (i) dialysis initiation to waitlisting; and (ii) waitlisting to deceased donor transplantation. Living donor transplantation and death were treated as competing events in cause‐specific hazard models, with censoring on 31 December 2023.

Multivariate models were fully adjusted for all covariates except diabetes. Diabetes status (yes/no) was recorded as a comorbidity independent of kidney failure aetiology, whereas diabetic kidney disease represented diabetes as the primary cause of kidney failure. Due to substantial overlap between diabetes and diabetic kidney disease, diabetes was excluded to minimise collinearity. Full univariate and multivariate model results are provided in Tables S1 and S2. cPRA was excluded from dialysis‐to‐waitlisting and waitlisting‐to‐death models due to lack of clinical relevance or availability.

Hazard ratios (HR) with 95% confidence intervals (CIs) were reported, with statistical significance defined as *p* ≤ 0.05. Interaction terms between sex and pre‐specified covariates were tested, with *p* ≤ 0.001 indicating significant effect modification. This more stringent threshold was selected a priori to reduce the likelihood of false‐positive findings arising from multiple interaction tests across several social and clinical domains, including covariates with multiple subgroup categories. Crude event rates per 100 000 person‐years (pys) were calculated for each outcome by sex and patient characteristics. Cumulative incidence plots for waitlisting and deceased donor transplantation were generated by sex and era (2006–2010, 2011–2015, 2016–2019, 2020–2023). Sensitivity analyses used Fine and Gray sub distribution hazard models for dialysis‐to‐waitlisting, waitlisting‐to‐deceased donor transplant and waitlisting‐to‐death outcomes. Missing data were minimal (BMI 2.1%, cPRA 0.1%, SEIFA 1.0%) and were handled using multiple imputation by chained equations [MICE]. Five imputed datasets were generated. Data were analysed using *STATA: Version 18.0* (StataCorp, College Station, TX, USA).

## 3 Results

### 3.1 Patient Demographics

A total of 47 884 patients with incident kidney failure who initiated dialysis in Australia between 2006 and 2023 were included, contributing 147 510 person‐years of follow‐up (Table 1). Patients were followed from dialysis initiation to kidney transplant waitlisting and, subsequently, receipt of a deceased donor kidney transplant (Figure S1). By study end, 23% were waitlisted and 47% died without ever being waitlisted (Table S3). Among those waitlisted, 78% received a transplant (deceased or living), 5% died while waiting and 17% remained on the waitlist at study end (Table S4).

**TABLE 1:** Characteristics of the Australian kidney failure population who are receiving dialysis (*n* = 47 884), and who entered the kidney transplant waitlist (*n* = 10 958) collected from 2006–2023, by sex.

	Total dialysis cohort			Waitlisted cohort		
	Female *n* (%)^a^	Male *n* (%)^a^	Total *n* (%)^a^	Female *n* (%)^a^	Male *n* (%)^a^	Total *n* (%)^a^
*N* (%)	17 948 (37.5)	29 936 (62.5)	47 884 (100.0)	4004 (36.5)	6954 (63.5)	10 958 (100.0)
Age at dialysis entry (years)						
< 18	229 (1.3)	343 (1.2)	572 (1.2)	141 (3.5)	215 (3.1)	356 (3.3)
18–45	3034 (16.9)	4099 (13.7)	7133 (14.9)	1373 (34.3)	2033 (29.2)	3406 (31.1)
46–65	7315 (40.8)	11 420 (38.1)	18 735 (39.1)	2121 (53.0)	3921 (56.4)	6042 (55.1)
> 65	7370 (41.0)	14 074 (47.0)	21 444 (44.8)	369 (9.2)	785 (11.3)	1154 (10.5)
Median [IQR]	62 [50–72]	64 [53–74]	63 [51–73]	50 [39–59]	52 [41–61]	52 [41–60]
Body mass index (BMI kg/m)						
Not obese (≤ 30)	10 862 (61.8)	19 305 (65.8)	30 167 (64.3)	2777 (70.6)	4776 (69.7)	7553 (70.0)
Obese (≥ 30)	6718 (38.2)	10 026 (34.2)	16 744 (35.7)	1156 (29.4)	2079 (30.3)	3235 (30.0)
Not collected	368	605	973	71	99	170
Ethnicity						
Australian & New Zealander/European	11 082 (61.7)	21 217 (70.9)	32 299 (67.5)	2422 (60.5)	4518 (65.0)	6940 (63.3)
Aboriginal & Torres Strait Islander	2936 (16.4)	2362 (7.9)	5298 (11.1)	297 (7.4)	386 (5.5)	683 (6.2)
Māori & Pacific Peoples	816 (4.5)	1063 (3.6)	1879 (3.9)	188 (4.7)	250 (3.6)	438 (4.0)
Asian	2115 (11.8)	3215 (10.7)	5330 (11.1)	752 (18.8)	1119 (16.1)	1871 (17.1)
Other	999 (5.6)	2079 (6.9)	3078 (6.4)	345 (8.6)	681 (9.8)	1026 (9.4)
Socio‐economic position						
Lowest 20% (most disadvantaged)	4916 (27.7)	7055 (23.8)	11 971 (25.3)	913 (23.0)	1488 (21.6)	2401 (22.1)
Highest 80% (least disadvantaged)	12 847 (72.3)	22 587 (76.2)	35 434 (74.7)	3049 (77.0)	5395 (78.4)	8444 (77.9)
Not collected	185	294	479	42	71	113
Cause of kidney failure						
Diabetic kidney disease	6876 (38.3)	11 009 (36.8)	17 885 (37.4)	696 (17.4)	1626 (23.4)	2322 (21.2)
Hypertension/renal vascular disease	2108 (11.7)	4609 (15.4)	6717 (14.0)	287 (7.2)	690 (9.9)	977 (8.9)
GN	3314 (18.5)	5995 (20.0)	9309 (19.4)	1307 (32.6)	2393 (34.4)	3700 (33.8)
Familial/hereditary kidney diseases	1429 (8.0)	1852 (6.2)	3281 (6.8)	772 (19.3)	964 (13.9)	1736 (15.8)
Tubulointerstitial disease	1832 (10.2)	2356 (7.9)	4188 (8.8)	527 (13.2)	683 (9.8)	1210 (11.1)
Other	2389 (13.3)	4115 (13.7)	6504 (13.6)	415 (10.3)	598 (8.6)	1013 (9.2)
Calculated panel reactive antibody (cPRA)						
0–19	2805 (79.4)	5889 (93.6)	8694 (88.5)	2393 (77.8)	5067 (93.2)	7460 (87.6)
20–84	504 (14.3)	374 (6.0)	878 (9.0)	467 (15.2)	341 (6.3)	808 (9.5)
85–94	110 (3.1)	11 (0.2)	121 (1.2)	106 (3.4)	11 (0.2)	117 (1.4)
95–98	77 (2.2)	11 (0.2)	88 (0.9)	75 (2.4)	11 (0.2)	86 (1.0)
≥ 99	37 (1.0)	3 (< 0.1)	40 (0.4)	36 (1.2)	3 (0.1)	39 (0.5)
Not collected	5	4	9	5	4	9
Not applicable	14 415	23 648	38 063	927	1521	2448
Year of dialysis entry						
2006–2010	3985 (22.2)	6361 (21.2)	10 346 (21.6)	904 (22.6)	1403 (20.2)	2307 (21.1)
2011–2015	4781 (26.6)	7827 (26.2)	12 608 (26.3)	1139 (28.4)	2073 (29.8)	3212 (29.3)
2016–2019	4376 (24.4)	7485 (25.0)	11 861 (24.8)	1140 (28.5)	2083 (29.9)	3223 (29.4)
2020–2023	4806 (26.8)	8263 (27.6)	13 069 (27.3)	821 (20.5)	1395 (20.1)	2216 (20.2)
Medical comorbidities						
Diabetes						
No	8935 (49.8)	15 004 (50.1)	23 939 (50.0)	3053 (76.2)	4779 (68.7)	7832 (71.5)
Type 1	775 (4.3)	1019 (3.4)	1749 (3.7)	163 (4.1)	214 (3.1)	377 (3.4)
Type 2	8238 (45.9)	13 913 (46.5)	22 151 (46.3)	788 (19.7)	1961 (28.2)	2749 (25.1)
Cerebrovascular disease						
Absent	15 862 (88.4)	26 087 (87.1)	41 949 (87.6)	3841 (95.9)	6633 (95.4)	10 474 (95.6)
Present	2086 (11.6)	3849 (12.9)	5935 (12.3)	163 (4.1)	321 (4.6)	484 (4.4)
Peripheral vascular disease						
Absent	14 389 (80.2)	22 713 (75.9)	37 102 (77.5)	3730 (93.2)	6272 (90.2)	10 002 (91.3)
Present	3559 (19.8)	7223 (24.1)	10 782 (22.5)	274 (6.8)	682 (9.8)	956 (8.7)
Coronary artery disease						
Absent	12 434 (69.3)	17 629 (58.9)	30 063 (62.8)	3597 (89.8)	5680 (81.7)	9277 (84.7)
Present	5514 (30.7)	12 307 (41.1)	17 821 (37.2)	407 (10.2)	1274 (18.3)	1681 (15.3)
Chronic lung disease						
Absent	15 240 (84.9)	25 269 (84.4)	40 509 (84.6)	3742 (93.5)	6461 (92.9)	10 203 (93.1)
Present	2708 (15.1)	4667 (15.6)	7373 (15.4)	262 (6.5)	493 (7.1)	755 (6.9)
Cancer pre‐dialysis						
Never had	15 493 (86.3)	24 562 (82.1)	40 055 (83.7)	3694 (92.3)	6383 (91.8)	10 077 (92.0)
Has ever had	2455 (13.7)	5374 (17.9)	7829 (16.3)	310 (7.7)	571 (8.2)	881 (8.0)
Comorbidity burden						
0	5037 (28.1)	7100 (23.7)	12 137 (25.4)	2395 (59.8)	3529 (50.8)	5924 (54.1)
1	5571 (31.0)	8420 (28.1)	13 991 (29.2)	1064 (26.6)	1999 (28.7)	3063 (28.0)
2	3853 (21.5)	6969 (23.3)	10 822 (22.6)	380 (9.5)	914 (13.1)	1294 (11.8)
≥ 3	3487 (19.4)	7447 (24.9)	10 934 (22.8)	165 (4.1)	512 (7.4)	677 (6.2)
Smoking						
Current	1889 (10.5)	3758 (12.5)	5647 (11.8)	267 (6.7)	696 (10.0)	963 (8.8)
Former	4790 (26.7)	13 492 (45.1)	18 282 (38.2)	989 (24.7)	2687 (38.6)	3676 (33.5)
Never	11 269 (62.8)	12 686 (42.4)	23 955 (50.0)	2748 (68.6)	3571 (51.4)	6319 (57.7)
Late Referral (seen < 3 months before 1st treatment)						
No	3358 (18.7)	5748 (19.2)	9106 (19.0)	614 (15.3)	1145 (16.5)	1759 (16.1)
Yes	14 590 (81.3)	24 188 (80.8)	38 778 (81.0)	3390 (84.7)	5809 (83.5)	9199 (83.9)
Treatment mode						
Haemodialysis	4713 (26.3)	7863 (26.3)	12 576 (26.3)	1729 (43.2)	2606 (37.5)	4335 (39.6)
Peritoneal dialysis	13 235 (73.7)	22 073 (73.7)	35 308 (73.7)	2275 (56.8)	4348 (62.5)	6623 (60.4)

^a^ Column percentages.

The dialysis cohort was predominantly male (62.5%), aged > 65 (44.8%) and identified as Australian & New Zealander/European (67.5%). Among those waitlisted, the majority were male (63.5%), aged 46–65 years (55.1%) and identified as Australian & New Zealander/European (63.3%) (Table 1).

### 3.2 Access to the Kidney Transplant Waitlist

#### 3.2.1 Waitlisting Rates

**FIGURE 2:**
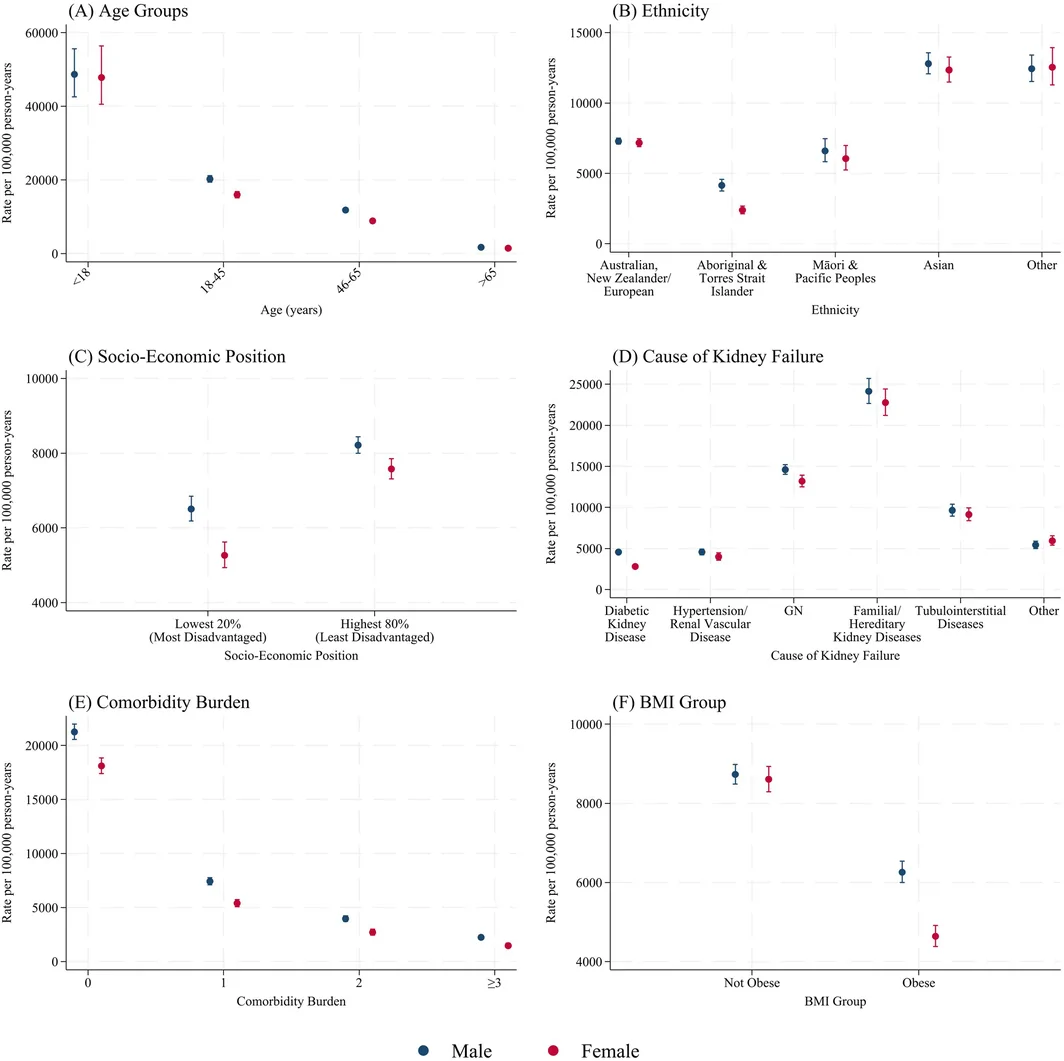
Rates of waitlisting per 100 000 person‐years.

Females had lower waitlisting rates than males (6884 vs. 7783 per 100 000 person‐years; Table S5). This sex‐based disparity was observed among 18–45‐ & 46–65‐year‐olds; Aboriginal & Torres Strait Islander peoples; all socio‐economic strata; all comorbidity burdens; obesity; and those whose cause of kidney failure was glomerulonephritis or hypertension/renal vascular disease (Figure 2).

#### 3.2.2 Likelihood of Waitlisting

In univariate analysis, female sex was associated with a 7% lower likelihood of waitlisting (HR 0.93, 95% CI 0.89–0.97). After multivariable adjustment, this disparity widened, with females having a 19% lower likelihood of waitlisting compared with males (aHR 0.81, 95% CI 0.77–0.84; Table 2).

**TABLE 2:** Hazard ratios for females compared with males, by outcome and model type.

Outcome	Model	Female; HR (95% CI)
Dialysis to waitlisting	Unadjusted	0.93 (0.89–0.97)
	Adjusted	0.81 (0.77–0.84)
Waitlisting to deceased donor transplant	Unadjusted	0.93 (0.88–0.97)
	Adjusted	1.04 (0.99–1.10)
Waitlisting to death before transplant	Unadjusted	0.78 (0.66–0.93)
	Adjusted	0.91 (0.76–1.08)

*Note:* HR > 1 indicates higher likelihood for females compared with males. Full univariate and multivariate model results are provided in Tables S1 and S2.

Significant interaction effects were observed between sex and ethnicity (*p* < 0.001), sex and comorbidity burden (*p* < 0.001), sex and cause of kidney failure (*p* < 0.001) and sex and BMI (*p* < 0.001), but not for age or socio‐economic position (Table 3).

**TABLE 3:** Multivariable association between hazard ratio (aHR) estimates from two‐way interaction terms with sex, and (A) Ethnicity, (B) Comorbidity burden, (C) Cause of kidney failure and (D) BMI groups, for dialysis patients to be waitlisted.

(A) Effect of female sex in ethnicity, *p* < 0.001	Univariate			Multivariate		
	HR	(95% CI)	*p*	aHR	(95% CI)	*p*
Australian & New Zealander/European	1.02	(0.97, 1.07)	0.501	0.85	(0.81, 0.90)	< 0.001
Aboriginal & Torres Strait Islander	0.59	(0.51, 0.69)	< 0.001	0.57	(0.49, 0.66)	< 0.001
Māori & Pacific Peoples	0.95	(0.78, 1.15)	0.582	0.80	(0.66, 0.96)	0.018
Asian	1.01	(0.92, 1.11)	0.850	0.77	(0.70, 0.85)	< 0.001
Other Ethnicities	1.05	(0.92, 1.20)	0.460	0.78	(0.68, 0.89)	< 0.001

(B) Effect of female sex in comorbidity burden, *p* < 0.001	Univariate			Multivariate		
	HR	(95% CI)	*p*	aHR	(95% CI)	*p*
0	0.90	(0.85, 0.95)	< 0.001	0.87	(0.83, 0.92)	< 0.001
1	0.76	(0.71, 0.82)	< 0.001	0.74	(0.69, 0.80)	< 0.001
2	0.72	(0.64, 0.81)	< 0.001	0.72	(0.63, 0.81)	< 0.001
≥ 3	0.67	(0.57, 0.80)	< 0.001	0.66	(0.56, 0.79)	< 0.001

(C) Effect of female sex in cause of kidney failure, *p* < 0.001	Univariate			Multivariate		
	HR	(95% CI)	*p*	aHR	(95% CI)	*p*
GN	0.95	(0.89, 1.01)	0.113	0.80	(0.74, 0.85)	< 0.001
Diabetic kidney disease	0.65	(0.60, 0.71)	< 0.001	0.61	(0.56, 0.67)	< 0.001
Hypertension/renal vascular disease	0.90	(0.79, 1.03)	0.140	0.79	(0.69, 0.91)	0.001
Familial/hereditary kidney disease	1.00	(0.91, 1.10)	0.964	0.92	(0.83, 1.01)	0.068
Tubulointerstitial disease	0.98	(0.87, 1.10)	0.731	1.02	(0.91, 1.14)	0.753
Other	1.15	(1.01, 1.30)	0.029	0.91	(0.81, 1.04)	0.156

(D) Effect of female sex in BMI groups, *p* < 0.001	Univariate			Multivariate		
	HR	(95% CI)	*p*	aHR	(95% CI)	*p*
Not obese	1.02	(0.98, 1.07)	0.327	0.86	(0.82, 0.90)	< 0.001
Obese	0.79	(0.74, 0.85)	0.000	0.69	(0.65, 0.75)	< 0.001

The largest ethnic disparity was observed among Aboriginal & Torres Strait Islander females, who had a 43% lower likelihood of waitlisting when compared to their male peers (aHR 0.57, 95% CI 0.49–0.66). Disparities were also evident among Asian females (23% lower; aHR 0.77, 95% CI 0.70–0.85), Māori & Pacific Peoples females (20% lower; aHR 0.80, 95% CI 0.66–0.96) and Australian & New Zealander/European females (15% lower; aHR 0.85, 95% CI 0.81–0.90) when compared to their respective male peers (Table 3A).

Sex disparities in accessing the kidney waitlist widened with increasing comorbidity burden, from 13% lower likelihood among females with no comorbidities (aHR 0.87, 95% CI 0.83–0.92) to 34% lower among those with ≥ 3 comorbidities (aHR 0.66, 95% CI 0.56–0.79), demonstrating a dose–response pattern (Table 3B).

Disparities were most pronounced among female patients with diabetic kidney disease, who were 39% less likely to be waitlisted (aHR 0.61, 95% CI 0.56–0.67) and remained evident among females with hypertension/renal vascular disease (21% lower; aHR 0.79, 95% CI 0.69–0.91) and glomerulonephritis (20% lower; aHR 0.80, 95% CI 0.74–0.85) (Table 3C).

Similarly, obese females were 31% less likely to be waitlisted compared to their obese male peers (aHR 0.69, 95% CI 0.65–0.75), whereas the disparity was smaller among non‐obese females compared with their non‐obese male peers (14% lower; aHR 0.86, 95% CI 0.82–0.90) (Table 3D).

### 3.3 Time to Waitlisting

Among waitlisted patients, the median time to waitlisting was 0.91 years (IQR: 0.40–1.86) (Table S3). Sex differences were statistically significant but small (females 0.92 vs. males 0.91 years; *p* < 0.001). Overall cumulative incidence curves showed no sex difference at 1 year; however, divergence emerged by 3 and 5 years following dialysis initiation (Figure S2A; Table S6A).

When stratified into year eras, fewer females were waitlisted at 3 and 5 years post dialysis initiation in 2011–2015 (3‐year CI: 19.2% vs. 22.1%; 5‐year CI: 21.8% vs. 24.7%) and at 3 years in 2016–2019 (21.8% vs. 24.7%), when compared to males (Figure S3; Table S7).

### 3.4 Access to Deceased Donor Transplantation

#### 3.4.1 Deceased Donor Transplant Rates

Among waitlisted patients, deceased donor transplant rates were lower in females than males (37 862 vs. 42 649 per 100 000 person‐years; Table S8). This disparity was observed across several demographic and clinical subgroups.

#### 3.4.2 Likelihood of Deceased Donor Transplant

In univariate analyses, female sex was associated with a 7% lower likelihood of receiving a deceased donor transplant. However, this association was no longer significant following multivariable adjustment (aHR 1.04, 95% CI 0.99–1.10; Table 2). Age, ethnicity, cPRA group, cause of kidney failure and BMI remained significant predictors.

### 3.5 Time to Deceased Donor Transplant After Waitlisting

The median time from waitlisting to deceased donor transplantation was 0.75 years [IQR: 0.22–2.05]. Waitlisted females had slightly longer median waiting times than waitlisted males (0.80 vs. 0.71 years; *p* = 0.001; Table S4), however, cumulative incidence did not differ significantly (Figure S2B; Table S6B), including across year eras (Table S9).

### 3.6 Mortality on the Waitlist

There was no significant difference in waitlist mortality rates by sex (Table S10) and no significant association between sex and waitlist mortality after adjustment (*p* = 0.286; Table 2). Among waitlisted patients who died, the median time to death was 3.73 years [IQR: 2.03–6.26], with females experiencing a longer time to death than males (4.52 years vs. 3.40 years; *p* < 0.001; Table S4).

## 4 Discussion

This national population‐based cohort study is the first in Australia to examine how sex and intersecting social and clinical disadvantage shape access to kidney transplantation. Our key findings confirm our central hypothesis that sex‐based disadvantage is not uniform, but is significantly modified by both social and clinical characteristics. Three findings stand out. First, females had a 19% lower adjusted rate of waitlisting compared with males, a disparity that widened after multivariable adjustment (from 7% unadjusted). Second, the average estimate concealed substantially greater disadvantage among females facing additional marginalisation, those of ethnic minority background, higher comorbidity burden, obesity and diabetic kidney disease. Third, once waitlisted, females and males had comparable adjusted rates of deceased donor transplantation, pinpointing the disparity to the pre‐waitlisting pathway.

Our finding that female sex is independently associated with reduced likelihood of waitlisting for dialysis patients aligns with prior studies from the United States [3, 4, 15, 16], Germany [7] and France [5, 6]. Our study extends this evidence by demonstrating where and for whom the disparity is greatest.

The intersectional dimension of this disadvantage was most evident by ethnicity. Pervasive inequities among ethnic minorities in access to transplantation are evident, with dialysis‐receiving Aboriginal & Torres Strait Islander patients being consistently less likely to be waitlisted than non‐Indigenous Australians [17]. Recent registry‐linked work capturing clinician‐reported reasons for non‐waitlisting suggests barriers occur at multiple stages, including delays in commencing or completing transplant work‐up, awaiting transplant assessment and both temporary and permanent contraindications [18]. Aboriginal & Torres Strait Islander people face well‐documented structural barriers, including geographic remoteness, institutional racism, high travel costs and limited access to culturally safe care [19]. These barriers do not operate in isolation. Rather, systems of power related to gender, race and socioeconomic position may interact to shape referral behaviour, clinical decision‐making and progression through transplant evaluation. In our cohort, Aboriginal & Torres Strait Islander females experienced a higher burden of treated kidney failure than their male peers (*n* = 2936 vs. *n* = 2362), yet fewer entered the kidney waitlist (aHR 0.57, 95% CI 0.49–0.66), suggesting that need alone does not translate into access. Cultural differences and rising reports of racial discrimination in healthcare settings are cited as primary reasons for disengagement from traditional healthcare [20]. Gendered family and community responsibilities may increase ‘time poverty’, limiting the capacity to complete the appointment‐intensive transplant work‐up required for waitlisting and may be compounded by remoteness and caring duties when travel to tertiary services is required. Other ethnic minority females in our cohort (Māori & Pacific Peoples, Asian, & Other) also experienced a larger relative reduction in access compared to their male counterparts than did Australian & New Zealander/European females, a gradient consistent with international evidence demonstrating that sex and ethnicity jointly shape referral, evaluation and waitlisting [21, 22]. Collectively, these observations suggest that intersecting systems of power act upstream of waitlisting, influencing who is referred, evaluated and ultimately deemed an appropriate transplant candidate.

The clinical dimension showed an equally consistent pattern and one that points to a common mechanism: a higher implicit health threshold for waitlisting among females. In our dialysis cohort, females had a comparatively healthier clinical profile, with 59.1% having one comorbidity or fewer compared with 51.8% of males. Yet females with no comorbidities, a group typically prioritised for transplantation, were still 13% less likely to be waitlisted than comparable males and this disparity widened in a dose–response manner with increasing comorbidity burden, reaching 34% among those with three or more comorbidities. Consistent with a higher threshold, waitlisted females survived longer on the waitlist than males (a difference of 1.12 years in median time to death), suggesting they were healthier at the point of waitlisting. Husain et al. reported the same phenomenon in the United States: even among dialysis patients with no medical comorbidities, female sex remained independently associated with lower access to waitlisting (adjusted subhazard ratio 0.92, 95% CI 0.90–0.94) [15]. Together with the widening of our overall estimate after adjustment, these findings indicate that while better health status improves the likelihood of waitlisting, even the healthiest females do not attain the access observed for males with comparable clinical status.

The same threshold effect was visible within specific clinical subgroups. Females with diabetic kidney disease experienced the greatest disadvantage, being 39% less likely to be waitlisted than their male peers. Although ANZDATA captures diabetic kidney disease as a single category encompassing both type‐1 and type‐2 diabetes, international studies that have stratified these conditions separately highlight important sex‐based inequities, reporting that females with type‐2 diabetes are 22%–27% less likely to be waitlisted than males [3, 4]. Diabetes carries a disproportionately higher relative risk of cardiovascular and kidney complications in females, alongside greater obesity and cardiometabolic burden [23, 24]—factors that may interact with transplant eligibility assessments and compound barriers to waitlisting for females with diabetic kidney disease. Similarly, females with obesity were 31% less likely to be waitlisted than males with obesity, indicating that weight‐related barriers, a recognised impediment to transplant referral and waitlisting [25], fall disproportionately on females. This aligns with global evidence that females with obesity are less likely to be deemed suitable during transplant referral and evaluation than their male peers [26, 27] and with the well‐documented observation that weight‐related stigma is directed more frequently and more severely at females [28]. Such stigma may operate precisely as our threshold hypothesis predicts—by raising implicit standards for referral and eligibility in ways that compound structural barriers.

If these disparities arise upstream of allocation, they should be largely independent of how healthcare is financed—and international comparisons bear this out. Australia operates a ‘hybrid’ healthcare model that combines a universal taxpayer‐funded public health insurance system (Medicare) with a private healthcare sector, providing publicly funded access to both dialysis and kidney transplantation. Comparable publicly funded models exist in France, Germany, Austria and Canada, yet sex disparities in access to waitlisting have been consistently reported across these settings [5, 6, 7, 8, 9], indicating that universal coverage alone is insufficient to eliminate them. The same disparity is evident in the United States [3, 4] where, despite an otherwise insurance‐based system dominated by private insurers, the Medicare End‐Stage Renal Disease Program provides near‐universal public funding for dialysis and transplantation. That inequities exist even under near‐universal, disease specific public coverage argues strongly that financing arrangements do not explain them. Notably, once patients are waitlisted, deceased donor allocation in these systems is largely algorithm‐driven and sex‐blind—consistent with our finding that the disparity attenuates after listing and reinforcing that the inequity resides in the human decision‐making stages of referral, evaluation and listing rather than in allocation itself.

This localisation carries direct implications for policy, because randomised evidence from similar disparities demonstrates that the pre‐waitlisting pathway is modifiable. In the United States, dialysis facility‐level quality improvement interventions have increased referral for transplant evaluation (the RaDIANT Community Study [29]) and attenuated racial disparities in waitlisting following allocation‐system reform (the ASCENT trial [30]), showing that structured, facility‐directed programs can impact the stages on the transplant pathway where the sex disparity arises. Notably, however, follow‐on analyses found that untargeted interventions may preferentially increase referral among already‐advantaged groups [31], underscoring that equity interventions must be explicitly targeted and their effects measured in a stratified fashion; otherwise, an intervention may widen the gap it was trying to close. No trial has yet specifically addressed sex‐based disparities in referral or waitlisting; the racial‐disparity trials provide the template and our findings identify the subgroups such trials should target.

A number of measures may help to reduce the disparities in transplant access we have found. First, automated, criteria‐triggered referral for transplant evaluation, functioning as an opt‐out rather than a discretionary step, would remove individual clinician judgement at the point where implicit sex‐based thresholds appear to operate. Second, ANZDATA's scope could be extended to capture referral and evaluation milestones, or a new registry established, paralleling the United States' Early Steps to Transplant Access Registry [32], enabling disparities to be localised and monitored rather than inferred and directly addressing the registry limitation we identify below. These structural measures should be complemented by service‐level supports with demonstrated relevance to the barriers we identify: patient navigator models, outreach and telehealth‐supported assessment to reduce the appointment and travel burden of work‐up, structured attention to logistical barriers such as caregiving responsibilities and geographic disadvantage and co‐designed referral pathways developed in partnership with transplant centres and affected communities.

This study possesses several strengths including the near‐complete national capture of dialysis, waitlisting, transplantation. However, there are limitations. First, ANZDATA records sex and gender together under a single variable (‘gender’), limiting our ability to separate biological sex‐related effects from gendered social influences on access. Second, as with all observational studies, residual confounding is possible. Factors such as health literacy, clinician‐level decision making, cultural attitudes towards health services, competing caregiving and family responsibilities, social support and patient preferences are not captured by ANZDATA. Third, the registry does not record referral practices or evaluation milestones, such as commencement of transplant workup, or reasons for non‐referral and non‐waitlisting, limiting our ability to determine precisely where disparities arise within the pre‐waitlisting pathway. Fourth, missing data required imputation, although proportions missing were low. Fifth, national eligibility criteria for waitlisting were amended in 2018, replacing the requirement for an ‘80% likelihood of survival at 5 years post‐transplant’ with a ‘high likelihood of significant benefit’ from transplantation [33]. Although this change may have increased the number of patients considered suitable for waitlisting, it was applied nationally and was not sex‐specific; consistent with this, sex differences in the cumulative incidence of waitlisting were already evident in eras preceding the change (Table S7), making it an unlikely explanation for the observed disparity. Finally, ANZDATA captures only individuals with kidney failure receiving kidney replacement therapy and therefore excludes those not considered suitable for dialysis; medically complex patients and socially disadvantaged patients may be underrepresented. If anything, this would be expected to bias our findings towards the null, as sex‐based inequities are known to emerge even earlier in the pathway, before dialysis initiation [34], implying that the disparities reported here may understate those across the broader population with kidney failure.

In conclusion, females with kidney failure in Australia experience substantially reduced access to the kidney transplant waitlist, with the greatest inequities affecting females who identify as Aboriginal & Torres Strait Islander, have a high comorbidity burden, diabetic kidney disease or are obese. Once waitlisted, females had similar rates of deceased donor transplantation, which places the disparity in the referral, evaluation and listing stages that come before it. The pattern across our analyses is that females had to be healthier than males to reach the same point of access. This points to a threshold that is applied unequally by sex and that sits in clinical decision‐making rather than in allocation. These decisions can be measured and audited and the evidence from comparable work on ethnic disparities shows they can be changed. Doing so for sex will require routine monitoring of who is referred and waitlisted and a willingness to act on what it shows.

## Author Contributions

Conceptualisation and study design: Melanie L. R Wyld. Data cleaning: Tennille L. Vitagliano. Biostatistical expertise: Nicole L. De La Mata. Coding: Tennille L. Vitagliano and Nicole L. De La Mata. Data analysis and interpretation of data: Tennille L. Vitagliano, Nicole L. De La Mata, Kate Wyburn and Melanie L. R Wyld. Clinical expertise: Peter S. Hsu, Stephen I. Alexander, Kate Wyburn and Melanie L. R Wyld. Manuscript preparation: Tennille L. Vitagliano, Nicole L. De La Mata, Peter S. Hsu, Stephen I. Alexander, Kate Wyburn and Melanie L. R Wyld. Funding and resources: Melanie L. R Wyld. All contributing authors have read, provided edits and approved the final version of the manuscript for submission.

## Funding

Author Melanie L. R Wyld has received research funding from The Jacquot Research Establishment Fellowship, Royal Australasian College of Physicians and is supported by a NHMRC Investigator Grant (2025/GNT2041699).

## Conflicts of Interest

The authors declare no conflicts of interest.

## Supporting information


**Figure S1:** Analysis models of the kidney failure population. Separate models depicting (A) dialysis to waitlisting and (B) waitlisting to deceased donor transplant, were analysed. Death and receipt of living donor transplant before primary outcome of each model was treated as a competing event. Both analyses share the transition state ‘waitlisted’.
**Table S1:** Univariate hazard ratio (HR) estimates associated with dialysis to waitlisting, waitlisting to deceased donor transplant and waitlisting to death before transplant.
**Table S2:** Multivariate hazard ratio (aHR) estimates associated with dialysis to waitlisting, waitlisting to deceased donor transplant and waitlisting to death before transplant.
**Table S3:** Summary of all incident patients, of all ages, with kidney failure who received dialysis in Australia (2006–2023).
**Table S4:** Summary of all incident patients, of all ages, with kidney failure who entered the kidney transplant waitlist in Australia (2006–2023).
**Table S5:** Rates per 100 000 person‐years for dialysis to waitlisting.
**Figure S2:** Cumulative incidence of time to waitlisting and transplant, by sex. Separate models measure (A) time to waitlisting from dialysis initiation and (B) time to receiving a deceased donor transplant after being waitlisted.
**Table S6:** 1‐, 3‐ and 5‐year cumulative incidence (CI) percentages of (A) time from dialysis to waitlisting and (B) time from waitlisting to deceased donor transplant, by sex.
**Figure S3:** Cumulative incidence of time to be waitlisted, by year eras and sex. Time periods display (A) 2006–2010, (B) 2011–2015, (C) 2016–2019 and (D) 2020–2023, from dialysis initiation.
**Table S7:** 1‐, 3‐ and 5‐year cumulative incidence (CI) percentages of time from dialysis to waitlisting, by year eras (A) 2006–2010, (B) 2011–2015, (C) 2016–2019 and (D) 2020–2023.
**Table S8:** Rates per 100 000 person‐years for waitlisting to receiving a deceased donor transplant.
**Table S9:** 1‐, 3‐ and 5‐year cumulative incidence (CI) percentages of time from waitlisting to receiving a deceased donor transplant, by year eras (A) 2006–2010, (B) 2011–2015, (C) 2016–2019 and (D) 2020–2023.
**Table S10:** Rates per 100 000 person‐years for dying before receiving a kidney transplant.

## Data Availability

The data that support the findings of this study are available on request from the corresponding author. The data are not publicly available due to privacy or ethical restrictions.
