# Supplementary material for: Sex and Intersectional Disparities in Access to Deceased Donor Kidney Transplantation: A 17‐Year Population‐Based Cohort Study
Source: Nephrology (Carlton). 2026 Sep 3;31(9):e70276. doi: 10.1111/nep.70276 (PMC13542340; doi:10.1111/nep.70276)
Supplement: Supplementary file 1 [file NEP-31-0-s001.docx]

**Supplementary** **Table of Contents**

[**Fig S1. Analysis models of the kidney failure population.** Separate models depicting (A) dialysis to waitlisting, and (B) waitlisting to deceased donor transplant, were analysed. Death and receipt of living donor transplant before primary outcome of each model was treated as a competing event. Both analyses share the transition state “waitlisted.”	2](#_Toc223130542)

[**Table S1.** Univariate hazard ratio (HR) estimates associated with dialysis to waitlisting, waitlisting to deceased donor transplant, and waitlisting to death before transplant.	3](#_Toc223130543)

[**Table S2.** Multivariate hazard ratio (aHR) estimates associated with dialysis to waitlisting, waitlisting to deceased donor transplant, and waitlisting to death before transplant.	5](#_Toc223130544)

[**Table S3.** Summary of all incident patients, of all ages, with kidney failure who received dialysis in Australia (2006-2023).	7](#_Toc223130545)

[**Table S4.** Summary of all incident patients, of all ages, with kidney failure who entered the kidney transplant waitlist in Australia (2006-2023).	8](#_Toc223130546)

[**Table S5.** Rates per 100,000 person-years for dialysis to waitlisting.	9](#_Toc223130547)

[**Fig S2.** Cumulative incidence of time to waitlisting and transplant, by sex. Separate models measure (A) time to waitlisting from dialysis initiation, and (B) time to receiving a deceased donor transplant after being waitlisted.	10](#_Toc223130548)

[**Table S6.** 1-, 3- and 5-year cumulative incidence (CI) percentages of (A) time from dialysis to waitlisting, and (B) time from waitlisting to deceased donor transplant, by sex.	11](#_Toc223130549)

[**Fig S3.** Cumulative incidence of time to be waitlisted, by year eras and sex. Time periods display (A) 2006-2010, (B) 2011-2015, (C) 2016-2019 and (D) 2020-2023, from dialysis initiation.	12](#_Toc223130550)

[**Table S7.** 1-, 3- and 5-year cumulative incidence (CI) percentages of time from dialysis to waitlisting, by year eras (A) 2006-2010, (B) 2011-2015, (C) 2016-2019, and (D) 2020-2023.	13](#_Toc223130551)

[**Table S8.** Rates per 100,000 person-years for waitlisting to receiving a deceased donor transplant.	15](#_Toc223130552)

[**Table S9.** 1-, 3- and 5-year cumulative incidence (CI) percentages of time from waitlisting to receiving a deceased donor transplant, by year eras (A) 2006-2010, (B) 2011-2015, (C) 2016-2019, and (D) 2020-2023.	16](#_Toc223130553)

[**Table S10.** Rates per 100,000 person-years for dying before receiving a kidney transplant.	18](#_Toc223130554)

### **Fig S1. Analysis models of the kidney failure population.** Separate models depicting (A) dialysis to waitlisting, and (B) waitlisting to deceased donor transplant, were analysed. Death and receipt of living donor transplant before primary outcome of each model was treated as a competing event. Both analyses share the transition state “waitlisted.”

### **Table S1.** Univariate hazard ratio (HR) estimates associated with dialysis to waitlisting, waitlisting to deceased donor transplant, and waitlisting to death before transplant.

| **Factors** | **Dialysis to Waitlisting** | | | **Waitlisting to Deceased Donor Transplant** | | | **Waitlisting to Death**  **Before Transplant** | | |
| --- | --- | --- | --- | --- | --- | --- | --- | --- | --- |
|  | **HR** | **(95% CI)** | **p-value** | **HR** | **(95% CI)** | **p-value** | **HR** | **(95% CI)** | **p-value** |
| **Sex** |  |  | **<0.001** |  |  | **0.001** |  |  | **0.004** |
| Male | **ref** |  |  | **ref** |  |  | **ref** |  |  |
| Female | 0.93 | (0.89,0.97) |  | 0.93 | (0.88,0.97) |  | 0.78 | (0.66,0.93) |  |
| **Age at Dialysis Entry (years)** |  |  | **<0.001** |  |  | **<0.001** |  |  | **<0.001** |
| <18 | 1.95 | (1.75,2.18) | <0.001 | 2.27 | (2.01,2.56) | <0.001 | 0.42 | (0.06,2.99) | 0.383 |
| 18-45 | **ref** |  |  | **ref** |  |  | **ref** |  |  |
| 46-65 | 0.58 | (0.56,0.61) | <0.001 | 1.01 | (0.96,1.06) | 0.628 | 1.91 | (1.54,2.38) | <0.001 |
| >65 | 0.09 | (0.08,0.10) | <0.001 | 0.99 | (0.91,1.07) | 0.735 | 3.71 | (2.85,4.83) | <0.001 |
| **Ethnicity** |  |  | **<0.001** |  |  | **<0.001** |  |  | **0.014** |
| Australian, New Zealander/European | **ref** |  |  | **ref** |  |  | **ref** |  |  |
| Aboriginal & Torres Strait Islander | 0.50 | (0.46,0.54) | <0.001 | 1.04 | (0.94,1.14) | 0.454 | 1.13 | (0.84,1.53) | 0.407 |
| Māori & Pacific Peoples | 0.94 | (0.86,1.04) | 0.234 | 0.81 | (0.72,0.91) | <0.001 | 1.07 | (0.76,1.50) | 0.691 |
| Asian | 1.71 | (1.63,1.80) | <0.001 | 0.75 | (0.71,0.80) | <0.001 | 0.80 | (0.64,0.99) | 0.044 |
| Other | 1.66 | (1.55,1.77) | <0.001 | 1.00 | (0.93,1.08) | 0.918 | 0.60 | (0.42,0.87) | 0.007 |
| **Socio-economic Position** |  |  | **<0.001** |  |  | **0.152** |  |  | **0.151** |
| Highest 80% (Least disadvantaged) | **ref** |  |  | **ref** |  |  | **ref** |  |  |
| Lowest 20% (Most disadvantaged) | 0.78 | (0.74,0.81) |  | 0.96 | (0.91,1.01) |  | 1.15 | (0.95,1.38) |  |
| **Diabetes** |  |  | **<0.001** |  |  | **<0.001** |  |  | **<0.001** |
| Absent | **ref** |  |  | **ref** |  |  | **ref** |  |  |
| Present | 0.34 | (0.33,0.36) |  | 0.86 | (0.81,0.90) |  | 2.96 | (2.51,3.48) |  |
| **Cerebrovascular Disease** |  |  | **<0.001** |  |  | **0.060** |  |  | **0.016** |
| Absent | **ref** |  |  | **ref** |  |  | **ref** |  |  |
| Present | 0.31 | (0.28,0.34) |  | 0.90 | (0.80,1.00) |  | 1.44 | (1.07,1.94) |  |
| **Peripheral Vascular Disease** |  |  | **<0.001** |  |  | **<0.001** |  |  | **<0.001** |
| Absent | **ref** |  |  | **ref** |  |  | **ref** |  |  |
| Present | 0.31 | (0.29,0.33) |  | 0.80 | (0.74,0.87) |  | 2.07 | (1.70,2.52) |  |
| **Coronary Artery Disease** |  |  | **<0.001** |  |  | **<0.001** |  |  | **<0.001** |
| Absent | **ref** |  |  | **ref** |  |  | **ref** |  |  |
| Present | 0.28 | (0.26,0.29) |  | 0.88 | (0.83,0.94) |  | 2.01 | (1.69,2.39) |  |
| **Chronic Lung Disease** |  |  | **<0.001** |  |  | **0.002** |  |  | **<0.001** |
| Absent | **ref** |  |  | **ref** |  |  | **ref** |  |  |
| Present | 0.39 | (0.36,0.42) |  | 0.87 | (0.79,0.95) |  | 1.56 | (1.23,1.98) |  |
| **Cancer Pre-Dialysis** |  |  | **<0.001** |  |  | **0.020** |  |  | **0.160** |
| Never Had | **ref** |  |  | **ref** |  |  | **ref** |  |  |
| Has ever had | 0.43 | (0.40,0.46) |  | 1.10 | (1.02,1.20) |  | 1.22 | (0.92,1.61) |  |
| **cPRA Group** |  |  |  |  |  | **<0.001** |  |  |  |
| 0-19 |  |  |  | **ref** |  |  |  |  |  |
| 20-84 |  |  |  | 0.71 | (0.66,0.77) | <0.001 |  |  |  |
| 85-94 |  |  |  | 0.74 | (0.61,0.89) | 0.001 |  |  |  |
| 95-98 |  |  |  | 0.68 | (0.54,0.84) | <0.001 |  |  |  |
| ≥99 |  |  |  | 0.41 | (0.30,0.57) | <0.001 |  |  |  |
| **Comorbidity Burden** |  |  | **<0.001** |  |  | **<0.001** |  |  | **<0.001** |
| 0 | **ref** |  |  | **ref** |  |  | **ref** |  |  |
| 1 | 0.35 | (0.33,0.36) | <0.001 | 0.96 | (0.91,1.01) | 0.113 | 2.65 | (2.14,3.28) | <0.001 |
| 2 | 0.18 | (0.17,0.20) | <0.001 | 0.88 | (0.82,0.95) | 0.001 | 3.59 | (2.85,4.54) | <0.001 |
| ≥3 | 0.10 | (0.09,0.11) | <0.001 | 0.74 | (0.67,0.81) | <0.001 | 4.39 | (3.43,5.62) | <0.001 |
| **Cause of Kidney Failure** |  |  | **<0.001** |  |  | **<0.001** |  |  | **<0.001** |
| GN | **ref** |  |  | **ref** |  |  | **ref** |  |  |
| Diabetic Kidney Disease | 0.27 | (0.26,0.29) | <0.001 | 0.86 | (0.80,0.91) | <0.001 | 3.49 | (2.82,4.33) | <0.001 |
| Hypertension/Renal Vascular Disease | 0.31 | (0.29,0.34) | <0.001 | 0.90 | (0.83,0.98) | 0.015 | 1.61 | (1.17,2.20) | 0.003 |
| Familial/Hereditary Kidney Diseases | 1.57 | (1.49,1.67) | <0.001 | 1.04 | (0.97,1.11) | 0.295 | 0.83 | (0.59,1.17) | 0.296 |
| Tubulointerstitial Disease | 0.68 | (0.64,0.73) | <0.001 | 1.09 | (1.01,1.18) | 0.025 | 1.39 | (0.99,1.96) | 0.056 |
| Other | 0.38 | (0.35,0.41) | <0.001 | 1.11 | (1.02,1.20) | 0.017 | 1.66 | (1.18,2.35) | 0.004 |
| **BMI Groups** |  |  | **<0.001** | **ref** |  | **<0.001** |  |  | **0.017** |
| Not Obese | **ref** |  |  | 1.11 | (1.06,1.17) |  | **ref** |  |  |
| Obese | 0.68 | (0.66,0.71) |  |  |  |  | 1.24 | (1.04,1.48) |  |
| **Smoking** |  |  | **<0.001** |  |  | **0.814** |  |  | **0.017** |
| Never | **ref** |  |  | **ref** |  |  | **ref** |  |  |
| Current | 0.58 | (0.54,0.62) | <0.001 | 1.02 | (0.95,1.11) | 0.543 | 1.19 | (0.91,1.54) | 0.205 |
| Former | 0.72 | (0.69,0.75) | <0.001 | 1.01 | (0.96,1.06) | 0.738 | 1.28 | (1.08,1.52) | 0.005 |
| **Late referral** |  |  | **<0.001** |  |  | **0.142** |  |  | **0.534** |
| No | **ref** |  |  | **ref** |  |  | **ref** |  |  |
| Yes | 0.80 | (0.76,0.84) |  | 1.05 | (0.99,1.11) |  | 0.93 | (0.75,1.16) |  |
| **Treatment Mode** |  |  | **<0.001** |  |  | **0.045** |  |  | **0.609** |
| Hemodialysis | **ref** |  |  | **ref** |  |  | **ref** |  |  |
| Peritoneal Dialysis | 2.03 | (1.95,2.11) |  | 0.95 | (0.91,1.00) |  | 1.04 | (0.89,1.23) |  |

### **Table S2.** Multivariate hazard ratio (aHR) estimates associated with dialysis to waitlisting, waitlisting to deceased donor transplant, and waitlisting to death before transplant.

| **Factors** | **Dialysis to Waitlisting** | | | **Waitlisting to Deceased Donor Transplant** | | | **Waitlisting to Death**  **Before Transplant** | | |
| --- | --- | --- | --- | --- | --- | --- | --- | --- | --- |
|  | **aHR** | **(95% CI)** | **p-value** | **aHR** | **(95% CI)** | **p-value** | **aHR** | **(95% CI)** | **p-value** |
| **Sex** |  |  | **<0.001** |  |  | **0.094** |  |  | **0.286** |
| Male | **ref** |  |  | **ref** |  |  | **ref** |  |  |
| Female | 0.81 | (0.77,0.84) |  | 1.04 | (0.99,1.10) |  | 0.91 | (0.76,1.08) |  |
| **Age at Dialysis Entry (years)** |  |  | **<0.001** |  |  | **<0.001** |  |  | **<0.001** |
| <18 | 0.92 | (0.82,1.03) | 0.142 | 1.95 | (1.72,2.21) | <0.001 | 0.61 | (0.08,4.41) | 0.623 |
| 18-45 | **ref** |  |  | **ref** |  |  | **ref** |  |  |
| 46-65 | 0.76 | (0.73,0.80) | <0.001 | 1.14 | (1.08,1.21) | <0.001 | 1.47 | (1.17,1.85) | 0.001 |
| >65 | 0.11 | (0.10,0.11) | <0.001 | 1.55 | (1.42,1.69) | <0.001 | 2.99 | (2.26,3.96) | <0.001 |
| **Ethnicity** |  |  | **<0.001** |  |  | **<0.001** |  |  | **0.029** |
| Australian, New Zealander/European | **ref** |  |  | **ref** |  |  | **ref** |  |  |
| Aboriginal & Torres Strait Islander | 0.44 | (0.40,0.48) | <0.001 | 1.30 | (1.18,1.44) | <0.001 | 0.84 | (0.61,1.15) | 0.277 |
| Māori & Pacific Peoples | 0.78 | (0.70,0.86) | <0.001 | 0.89 | (0.78,1.00) | 0.049 | 0.76 | (0.54,1.08) | 0.126 |
| Asian | 1.22 | (1.15,1.29) | <0.001 | 0.80 | (0.75,0.86) | <0.001 | 0.76 | (0.60,0.96) | 0.019 |
| Other | 1.30 | (1.22,1.39) | <0.001 | 0.96 | (0.89,1.04) | 0.354 | 0.64 | (0.44,0.93) | 0.020 |
| **Socio-economic Position** |  |  | **<0.001** |  |  | **0.380** |  |  | **0.104** |
| Highest 80% (Least disadvantaged) | **ref** |  |  | **ref** |  |  | **ref** |  |  |
| Lowest 20% (Most disadvantaged) | 0.81 | (0.78,0.85) |  | 1.02 | (0.97,1.08) |  | 1.17 | (0.97,1.42) |  |
| **Cerebrovascular Disease** |  |  | **<0.001** |  |  | **0.553** |  |  | **0.044** |
| Absent | **ref** |  |  | **ref** |  |  | **ref** |  |  |
| Present | 0.76 | (0.69,0.85) |  | 1.04 | (0.91,1.19) |  | 0.70 | (0.49,0.99) |  |
| **Peripheral Vascular Disease** |  |  | **0.062** |  |  | **0.272** |  |  | **0.140** |
| Absent | **ref** |  |  | **ref** |  |  | **ref** |  |  |
| Present | 0.91 | (0.82,1.01) |  | 0.93 | (0.82,1.06) |  | 0.78 | (0.56,1.09) |  |
| **Coronary Artery Disease** |  |  | **0.001** |  |  | **0.716** |  |  | **0.215** |
| Absent | **ref** |  |  | **ref** |  |  | **ref** |  |  |
| Present | 0.86 | (0.79,0.94) |  | 0.98 | (0.88,1.09) |  | 0.82 | (0.61,1.12) |  |
| **Chronic Lung Disease** |  |  | **0.229** |  |  | **0.080** |  |  | **0.227** |
| Absent | **ref** |  |  | **ref** |  |  | **ref** |  |  |
| Present | 0.94 | (0.86,1.04) |  | 0.90 | (0.79,1.01) |  | 0.82 | (0.59,1.13) |  |
| **Cancer Pre-Dialysis** |  |  | **0.070** |  |  | **0.899** |  |  | **0.009** |
| Never Had | **ref** |  |  | **ref** |  |  | **ref** |  |  |
| Has ever had | 0.92 | (0.84,1.01) |  | 0.99 | (0.88,1.12) |  | 0.60 | (0.41,0.88) |  |
| **cPRA Group** |  |  |  |  |  | **<0.001** |  |  |  |
| 0-19 |  |  |  | **ref** |  |  |  |  |  |
| 20-84 |  |  |  | 0.71 | (0.66,0.77) | <0.001 |  |  |  |
| 85-94 |  |  |  | 0.71 | (0.58,0.86) | <0.001 |  |  |  |
| 95-98 |  |  |  | 0.65 | (0.52,0.81) | <0.001 |  |  |  |
| ≥99 |  |  |  | 0.43 | (0.31,0.60) | <0.001 |  |  |  |
| **Comorbidity Burden** |  |  | **<0.001** |  |  | **0.022** |  |  | **<0.001** |
| 0 | **ref** |  |  | **ref** |  |  | **ref** |  |  |
| 1 | 0.64 | (0.59,0.68) | <0.001 | 1.14 | (1.04,1.25) | 0.005 | 2.11 | (1.57,2.83) | <0.001 |
| 2 | 0.46 | (0.40,0.52) | <0.001 | 1.19 | (1.01,1.41) | 0.040 | 3.14 | (1.91,5.15) | <0.001 |
| ≥3 | 0.35 | (0.28,0.44) | <0.001 | 1.22 | (0.92,1.62) | 0.175 | 4.57 | (2.12,9.87) | <0.001 |
| **Cause of Kidney Failure** |  |  | **<0.001** |  |  | **0.015** |  |  | **0.001** |
| GN | **ref** |  |  | **ref** |  |  | **ref** |  |  |
| Diabetic Kidney Disease | 0.64 | (0.60,0.70) | <0.001 | 1.10 | (1.00,1.21) | 0.060 | 1.78 | (1.31,2.42) | <0.001 |
| Hypertension/Renal Vascular Disease | 0.68 | (0.63,0.73) | <0.001 | 0.98 | (0.90,1.07) | 0.648 | 1.17 | (0.85,1.61) | 0.349 |
| Familial/Hereditary Kidney Diseases | 1.39 | (1.31,1.47) | <0.001 | 1.00 | (0.93,1.07) | 0.907 | 0.78 | (0.55,1.11) | 0.164 |
| Tubulointerstitial Disease | 0.81 | (0.76,0.86) | <0.001 | 1.01 | (0.93,1.09) | 0.799 | 1.28 | (0.90,1.81) | 0.162 |
| Other | 0.59 | (0.55,0.63) | <0.001 | 1.14 | (1.05,1.24) | 0.002 | 1.45 | (1.02,2.06) | 0.039 |
| **BMI Groups** |  |  | **<0.001** |  |  | **<0.001** |  |  | **0.917** |
| Not Obese | **ref** |  |  | **ref** |  |  | **ref** |  |  |
| Obese | 0.72 | (0.69,0.75) |  | 1.13 | (1.07,1.19) |  | 0.99 | (0.82,1.19) |  |
| **Smoking** |  |  | **<0.001** |  |  | **0.002** |  |  | **0.162** |
| Never | **ref** |  |  | **ref** |  |  | **ref** |  |  |
| Current | 0.47 | (0.44,0.51) | <0.001 | 1.15 | (1.06,1.24) | 0.001 | 1.31 | (0.99,1.73) | 0.057 |
| Former | 0.94 | (0.91,0.99) | 0.009 | 0.99 | (0.94,1.04) | 0.584 | 1.06 | (0.89,1.27) | 0.529 |
| **Late referral** |  |  | **<0.001** |  |  | **0.122** |  |  | **0.970** |
| No | **ref** |  |  | **ref** |  |  | **ref** |  |  |
| Yes | 0.72 | (0.69,0.76) |  | 1.05 | (0.99,1.12) |  | 1.00 | (0.79,1.25) |  |
| **Treatment Mode** |  |  | **<0.001** |  |  | **0.323** |  |  | **0.213** |
| Hemodialysis | **ref** |  |  | **ref** |  |  | **ref** |  |  |
| Peritoneal Dialysis | 1.52 | (1.46,1.58) |  | 1.02 | (0.98,1.07) |  | 1.12 | (0.94,1.32) |  |

### **Table S3.** Summary of all incident patients, of all ages, with kidney failure who received dialysis in Australia (2006-2023).

| **Dialysis Cohort** | **Female** | **Male** | **Total** | **Female** | **Male** | **All** |  |
| --- | --- | --- | --- | --- | --- | --- | --- |
|  | **n (%) ^α^** | **n (%) ^α^** | **n (%) ^α^** | **Median time to event, years [IQR]** | | | **P-value*** |
| Waitlisted | 4,004  (22%) | 6,954  (23%) | 10,958  (23%) | 0.92  [0.37-1.90] | 0.91  [0.41-1.84] | 0.91  [0.40-1.86] | <0.001 |
| Living donor transplant without being waitlisted | 468  (3%) | 881  (3%) | 1,349  (3%) | 0.70  [0.32-1.26] | 0.72  [0.32-1.34] | 0.71  [0.32-1.30] | 0.015 |
| Death without being waitlisted | 8,370  (47%) | 14,249  (48%) | 22,619  (47%) | 2.97  [1.28-5.46] | 2.85  [1.28-5.14] | 2.90  [1.28-5.25] | <0.001 |
| Censored** | 5,106  (28%) | 7,852  (26%) | 12,958  (27%) | 2.96  [1.19-5.83] | 2.60  [1.11-5.09] | 2.72  [1.14-5.38] | 0.234 |
| **Total** | 17,948 | 29,936 | 47,884 |  |  |  |  |

**^α^** Column percentages
*P-value: Log-rank test by sex
**Censored patients: remained on KRT at the conclusion of the study period (31 Dec 2023).

### **Table S4.** Summary of all incident patients, of all ages, with kidney failure who entered the kidney transplant waitlist in Australia (2006-2023).

| **Waitlisted Cohort** | **Female** | **Male** | **Total** | **Female** | **Male** | **All** |  |
| --- | --- | --- | --- | --- | --- | --- | --- |
|  | **n (%) ^α^** | **n (%) ^α^** | **n (%) ^α^** | **Median time to event, years [IQR]** | | | **P-value*** |
| Deceased donor transplant | 2,784  (70%) | 4,939  (71%) | 7,723  (70%) | 0.80  [0.23-2.12] | 0.71  [0.21-2.01] | 0.75  [0.22-2.05] | 0.001 |
| Living donor transplant | 298  (7%) | 498  (7%) | 796  (7%) | 0.79  [0.37-1.33] | 0.80  [0.42-1.47] | 0.80  [0.40-1.43] | 0.948 |
| Died waiting to be transplanted | 221  (6%) | 380  (6%) | 601  (6%) | 4.52  [2.35-7.29] | 3.40  [1.74-5.81] | 3.73  [2.03-6.26] | 0.004 |
| Censored** | 701  (17%) | 1,137  (16%) | 1,838  (17%) | 1.58  [0.57-3.35] | 1.59  [0.68-3.06] | 1.59  [0.63-3.16] | 0.356 |
| **Total** | 4,004 | 6,954 | 10,958 |  |  |  |  |

**^α^** Column percentages
*P-value: Log-rank test by sex
**Censored patients: remained on KRT at the conclusion of the study period (31 Dec 2023).

### **Table S5.** Rates per 100,000 person-years for dialysis to waitlisting.

| **Rates per 100,000 Person-Years** | **Dialysis to Waitlisting** | | | | | | |
| --- | --- | --- | --- | --- | --- | --- | --- |
|  | **Female** | | | **Male** | | |  |
|  | **Count** | **Rate** | **95% CI** | **Count** | **Rate** | **95% CI** |  |
| **Sex** | 4,004 | 6,884 | (6,674-7,101) | 6,954 | 7,783 | (7,602-7,968) |  |
| **Age at dialysis initiation (years)** |  |  |  |  |  |  |  |
| <18 | 141 | 47,817 | (40,541-56,398) | 215 | 48,695 | (42,602-55,659) |  |
| 18-45 | 1,373 | 15,966 | (15,144-16,834) | 2,033 | 20,268 | (19,406-21,168) |  |
| 46-65 | 2,121 | 8,876 | (8,506-9,261) | 3,921 | 11,825 | (11,460-12,201) |  |
| >65 | 369 | 1,454 | (1,313-1,611) | 785 | 1,717 | (1,601-1,842) |  |
| **Ethnicity** |  |  |  |  |  |  |  |
| Australian, New Zealander/European | 2,422 | 7,171 | (6,891-7,462) | 4,518 | 7,285 | (7,075-7,500) |  |
| Aboriginal & Torres Strait Islander | 297 | 2,387 | (2,131-2,675) | 386 | 4,145 | (3,752-4,580) |  |
| Māori & Pacific Peoples | 188 | 6,047 | (5,241-6,976) | 250 | 6,596 | (5,827-7,467) |  |
| Asian | 752 | 12,348 | (11,496-13,263) | 1,119 | 12,796 | (12,068-13,569) |  |
| Other | 345 | 12,545 | (11,289-13,941) | 681 | 12,432 | (11,533-13,402) |  |
| **Socio-Economic Position** |  |  |  |  |  |  |  |
| Lowest 20% (Most disadvantaged) | 913 | 5,266 | (4,935-5,619) | 1,488 | 6,506 | (6,184-6,845) |  |
| Highest 80% (Least disadvantaged) | 3,049 | 7,578 | (7,313-7,851) | 5,395 | 8,215 | (7,998-8,437) |  |
| **Comorbidity Burden** |  |  |  |  |  |  |  |
| 0 | 2,395 | 18,104 | (17,394-18,844) | 3,529 | 21,257 | (20,567-21,970) |  |
| 1 | 1,064 | 5,402 | (5,087-5,736) | 1,999 | 7,431 | (7,112-7,764) |  |
| 2 | 380 | 2,716 | (2,456-3,003) | 914 | 3,965 | (3,716-4,230) |  |
| ≥3 | 165 | 1,467 | (1,260-1,709) | 512 | 2,247 | (2,060-2,450) |  |
| **Cause of Kidney Failure** |  |  |  |  |  |  |  |
| Diabetic Kidney Disease | 696 | 2,795 | (2,595-3,010) | 1,626 | 4,553 | (4,337-4,779) |  |
| Hypertension/Renal Vascular Disease | 287 | 3,990 | (3,554-4,480) | 690 | 4,565 | (4,237-4,919) |  |
| GN | 1,307 | 13,190 | (12,494-13,924) | 2,393 | 14,598 | (14,025-15,195) |  |
| Familial/Hereditary Kidney Diseases | 772 | 22,762 | (21,212-24,426) | 964 | 24,130 | (22,654-25,703) |  |
| Tubulointerstitial Disease | 527 | 9,127 | (8,380-9,940) | 683 | 9,637 | (8,940-10,387) |  |
| Other | 415 | 5,934 | (5,390-6,533) | 598 | 5,417 | (5,000-5,869) |  |
| **BMI Groups** |  |  |  |  |  |  |  |
| Not Obese | 2,777 | 8,608 | (8,294-8,935) | 4,776 | 8,731 | (8,487-8,982) |  |
| Obese | 1,156 | 4,639 | (4,379-4,914) | 2,079 | 6,259 | (5,996-6,534) |  |

### **Fig S2.** Cumulative incidence of time to waitlisting and transplant, by sex. Separate models measure (A) time to waitlisting from dialysis initiation, and (B) time to receiving a deceased donor transplant after being waitlisted.

### **Table S6.** 1-, 3- and 5-year cumulative incidence (CI) percentages of (A) time from dialysis to waitlisting, and (B) time from waitlisting to deceased donor transplant, by sex.

| **Cumulative Incidence (%)** | (A) **Cumulative Incidence of Time from  Dialysis to Waitlisting** | | | |
| --- | --- | --- | --- | --- |
|  | **Female** | | **Male** | |
|  | **CI (%)** | **95% CI** | **CI (%)** | **95% CI** |
| **1-year** |  |  |  |  |
| Waitlisted | 11.9 | (11.5-12.4) | 12.5 | (12.1-12.8) |
| Died before waitlisted | 9.3 | (8.9-9.8) | 9.6 | (9.2-9.9) |
| Received living donor transplant | 1.8 | (1.6-2.0) | 1.8 | (1.7-2.0) |
| End of follow-up | 6.2 | (5.8-6.5) | 6.1 | (5.8-6.3) |
| **3-years** |  |  |  |  |
| Waitlisted | 19.2 | (18.7-19.8) | 20.6 | (20.1-21.0) |
| Died before waitlisted | 23.5 | (22.9-24.1) | 24.8 | (24.3-25.3) |
| Received living donor transplant | 2.5 | (2.3-2.8) | 2.8 | (2.7-3.0) |
| End of follow-up | 14.4 | (13.9-14.9) | 14.5 | (14.1-14.9) |
| **5-years** |  |  |  |  |
| Waitlisted | 21.1 | (20.5-21.7) | 22.3 | (21.8-22.8) |
| Died before waitlisted | 33.3 | (32.6-34.0) | 35.1 | (34.6-35.7) |
| Received living donor transplant | 2.6 | (2.4-2.8) | 2.9 | (2.8-3.1) |
| End of follow-up | 19.7 | (19.1-20.3) | 19.5 | (19.1-20.0) |

| **Cumulative Incidence (%)** | (B) **Cumulative Incidence of Time from Waitlisting to Deceased Donor Transplant** | | | |  |
| --- | --- | --- | --- | --- | --- |
|  | **Female** | | **Male** | |  |
|  | **CI (%)** | **95% CI** | **CI (%)** | **95% CI** | |
| **1-year** |  |  |  |  | |
| Received deceased donor transplant | 39.0 | (37.5-40.5) | 40.5 | (39.4-41.7) | |
| Died before transplant | 0.5 | (0.3-0.8) | 0.6 | (0.4-0.7) | |
| Received living donor transplant | 4.7 | (4.1-5.4) | 4.3 | (3.9-4.8) | |
| End of follow-up | 6.4 | (5.6-7.2) | 5.6 | (5.0-6.1) | |
| **3-years** |  |  |  |  | |
| Received deceased donor transplant | 58.6 | (57.1-60.1) | 60.7 | (59.5-61.8) | |
| Died before transplant | 1.8 | (1.4-2.3) | 2.4 | (2.1-2.8) | |
| Received living donor transplant | 6.9 | (6.1-7.7) | 6.8 | (6.2-7.4) | |
| End of follow-up | 12.6 | (11.6-13.6) | 12.1 | (11.4-12.9) | |
| **5-years** |  |  |  |  | |
| Received deceased donor transplant | 66.8 | (65.3-68.2) | 68.8 | (67.7-69.9) | |
| Died before transplant | 3.1 | (2.6-3.7) | 3.7 | (3.3-4.2) | |
| Received living donor transplant | 7.3 | (6.5-8.2) | 7.1 | (6.5-7.7) | |
| End of follow-up | 15.2 | (14.1-16.3) | 14.7 | (13.9-15.5) | |

### **Fig S3.** Cumulative incidence of time to be waitlisted, by year eras and sex. Time periods display (A) 2006-2010, (B) 2011-2015, (C) 2016-2019 and (D) 2020-2023, from dialysis initiation.

### **Table S7.** 1-, 3- and 5-year cumulative incidence (CI) percentages of time from dialysis to waitlisting, by year eras (A) 2006-2010, (B) 2011-2015, (C) 2016-2019, and (D) 2020-2023.

| **Cumulative Incidence (%)** | (A) **Cumulative incidence of time from dialysis to waitlisting for year era 2006-2010** | | | |  |
| --- | --- | --- | --- | --- | --- |
|  | **Female** | | **Male** | |  |
|  | **CI (%)** | **95% CI** | **CI (%)** | **95% CI** | |
| **1-year** |  |  |  |  | |
| Waitlisted | 11.2 | (10.2-12.2) | 11.2 | (10.5-12.0) | |
| Died before waitlisted | 12.0 | (11.0-13.0) | 11.4 | (10.6-12.2) | |
| Received living donor transplant | 2.4 | (2.0-3.0) | 2.7 | (2.4-3.2) | |
| End of follow-up | NA |  | NA |  | |
| **3-years** |  |  |  |  | |
| Waitlisted | 18.5 | (17.4-19.8) | 18.6 | (17.6-19.5) | |
| Died before waitlisted | 29.4 | (28.0-30.8) | 29.7 | (28.6-30.9) | |
| Received living donor transplant | 3.9 | (3.3-4.5) | 4.6 | (4.1-5.2) | |
| End of follow-up | NA |  | NA |  | |
| **5-years** |  |  |  |  | |
| Waitlisted | 20.5 | (19.3-21.8) | 20.5 | (19.5-21.5) | |
| Died before waitlisted | 42.4 | (40.9-44.0) | 43.4 | (42.2-44.7) | |
| Received living donor transplant | 4.0 | (3.4-4.7) | 4.9 | (4.4-5.4) | |
| End of follow-up | NA |  | NA |  | |

| **Cumulative Incidence (%)** | (B) **Cumulative incidence of time from dialysis to waitlisting for year era  2011-2015** | | | |  |
| --- | --- | --- | --- | --- | --- |
|  | **Female** | | **Male** | |  |
|  | **CI (%)** | **95% CI** | **CI (%)** | **95% CI** | |
| **1-year** |  |  |  |  | |
| Waitlisted | 11.2 | (10.3-12.1) | 12.6 | (11.9-13.3) | |
| Died before waitlisted | 9.2 | (8.4-10.0) | 10.3 | (9.7-11.0) | |
| Received living donor transplant | 2.2 | (1.8-2.6) | 2.3 | (2.0-2.7) | |
| End of follow-up | NA |  | NA |  | |
| **3-years** |  |  |  |  | |
| Waitlisted | 19.2 | (18.1-20.3) | 22.1 | (21.2-23.0) | |
| Died before waitlisted | 25.3 | (24.0-26.5) | 27.5 | (26.5-28.5) | |
| Received living donor transplant | 3.0 | (2.5-3.5) | 3.5 | (3.1-3.9) | |
| End of follow-up | NA |  | NA |  | |
| **5-years** |  |  |  |  | |
| Waitlisted | 21.8 | (20.7-23.0) | 24.7 | (23.8-25.7) | |
| Died before waitlisted | 39.0 | (37.7-40.4) | 41.0 | (39.9-42.1) | |
| Received living donor transplant | 3.0 | (2.6-3.6) | 3.7 | (3.3-4.1) | |
| End of follow-up | NA |  | NA |  | |

| **Cumulative Incidence (%)** | (C) **Cumulative incidence of time from dialysis to waitlisting for year era  2016-2019** | | | |  |
| --- | --- | --- | --- | --- | --- |
|  | **Female** | | **Male** | |  |
|  | **CI (%)** | **95% CI** | **CI (%)** | **95% CI** | |
| **1-year** |  |  |  |  | |
| Waitlisted | 13.9 | (12.8-14.9) | 14.7 | (13.9-15.5) | |
| Died before waitlisted | 9.4 | (8.6-10.3) | 8.9 | (8.2-9.5) | |
| Received living donor transplant | 1.5 | (1.2-1.9) | 1.5 | (1.2-1.8) | |
| End of follow-up | NA |  | NA |  | |
| **3-years** |  |  |  |  | |
| Waitlisted | 22.7 | (21.5-23.9) | 25.0 | (24.0-26.0) | |
| Died before waitlisted | 24.4 | (23.1-25.7) | 25.9 | (24.9-26.9) | |
| Received living donor transplant | 2.1 | (1.7-2.6) | 2.2 | (1.9-2.5) | |
| End of follow-up | NA |  | NA |  | |
| **5-years** |  |  |  |  | |
| Waitlisted | 25.3 | (24.0-26.6) | 27.3 | (26.3-28.3) | |
| Died before waitlisted | 36.6 | (35.2-38.1) | 40.1 | (39.0-41.2) | |
| Received living donor transplant | 2.1 | (1.7-2.6) | 2.2 | (1.9-2.6) | |
| End of follow-up | 10.0 | (9.1-10.9) | 8.6 | (7.9-9.2) | |

| **Cumulative Incidence (%)** | (D) **Cumulative incidence of time from dialysis to waitlisting for year era  2020-2023** | | | |  |
| --- | --- | --- | --- | --- | --- |
|  | **Female** | | **Male** | |  |
|  | **CI (%)** | **95% CI** | **CI (%)** | **95% CI** | |
| **1-year** |  |  |  |  | |
| Waitlisted | 11.6 | (10.7-12.5) | 11.3 | (10.6-12.0) | |
| Died before waitlisted | 7.3 | (6.6-8.0) | 8.0 | (7.5-8.6) | |
| Received living donor transplant | 1.0 | (0.8-1.3) | 1.0 | (0.8-1.3) | |
| End of follow-up | 23.0 | (21.8-24.2) | 21.9 | (21.1-22.8) | |
| **3-years** |  |  |  |  | |
| Waitlisted | 16.8 | (15.7-17.8) | 16.7 | (15.9-17.5) | |
| Died before waitlisted | 16.2 | (15.1-17.2) | 17.5 | (16.7-18.4) | |
| Received living donor transplant | 1.4 | (1.1-1.8) | 1.4 | (1.2-1.7) | |
| End of follow-up | 53.9 | (52.4-55.3) | 52.6 | (51.6-53.7) | |
| **5-years** |  |  |  |  | |
| Waitlisted | 17.1 | (16.0-18.2) | 16.9 | (16.1-17.7) | |
| Died before waitlisted | 17.0 | (16.0-18.1) | 18.7 | (17.9-19.6) | |
| Received living donor transplant | 1.4 | (1.1-1.8) | 1.4 | (1.2-1.7) | |
| End of follow-up | 64.5 | (63.1-65.8) | 63.0 | (62.0-64.0) | |

### **Table S8.** Rates per 100,000 person-years for waitlisting to receiving a deceased donor transplant.

| **Rates per 100,000 Person-Years** | **Waitlisting to Deceased Donor Transplant** | | | | | |
| --- | --- | --- | --- | --- | --- | --- |
|  | **Female** | | | **Male** | | |
|  | **Count** | **Rate** | **95% CI** | **Count** | **Rate** | **95% CI** |
| **Sex** | 2,784 | 37,862 | (36,481-39,295) | 4,939 | 42,649 | (41,476-43,855) |
| **Age at dialysis initiation (years)** |  |  |  |  |  |  |
| <18 | 118 | 117,492 | (98,095-140,724) | 190 | 115,599 | (100,277-133,262) |
| 18-45 | 948 | 37,705 | (35,379-40,183) | 1,433 | 41,522 | (39,427-43,729) |
| 46-65 | 1,477 | 35,949 | (34,162-37,830) | 2,828 | 41,911 | (40,394-43,484) |
| >65 | 241 | 38,268 | (33,729-43,418) | 488 | 40,084 | (36,680-43,802) |
| **Ethnicity** |  |  |  |  |  |  |
| Australian, New Zealander/European | 1,704 | 39,864 | (38,016-41,803) | 3,240 | 45,916 | (44,362-47,525) |
| Aboriginal & Torres Strait Islander | 204 | 42,779 | (37,294-49,071) | 285 | 44,110 | (39,275-49,541) |
| Māori & Pacific Peoples | 125 | 30,187 | (25,333-35,971) | 173 | 35,648 | (30,713-41,376) |
| Asian | 494 | 30,269 | (27,714-33,060) | 744 | 32,880 | (30,600-35,329) |
| Other | 257 | 46,261 | (40,937-52,277) | 497 | 43,977 | (40,275-48,018) |
| **Socio-Economic Position** |  |  |  |  |  |  |
| Lowest 20% (Most disadvantaged) | 628 | 36,112 | (33,395-39,050) | 1,053 | 41,287 | (38,867-43,858) |
| Highest 80% (Least disadvantaged) | 2,128 | 38,375 | (36,779-40,041) | 3,833 | 42,841 | (41,506-44,219) |
| **Comorbidity Burden** |  |  |  |  |  |  |
| 0 | 1,749 | 41,371 | (39,477-43,356) | 2,590 | 44,472 | (42,792-46,218) |
| 1 | 710 | 36,474 | (33,887-39,258) | 1,406 | 43,930 | (41,693-46,287) |
| 2 | 227 | 28,166 | (24,730-32,079) | 628 | 41,200 | (38,100-44,551) |
| ≥3 | 98 | 26,281 | (21,560-32,035) | 315 | 30,526 | (27,334-34,090) |
| **Cause of Kidney Failure** |  |  |  |  |  |  |
| Diabetic Kidney Disease | 408 | 28,562 | (25,920-31,472) | 1,070 | 38,072 | (35,857-40,422) |
| Hypertension/Renal Vascular Disease | 198 | 34,171 | (29,728-39,279) | 480 | 37,106 | (33,931-40,579) |
| GN | 931 | 37,484 | (35,152-39,971) | 1,731 | 44,167 | (42,135-46,297) |
| Familial/Hereditary Kidney Diseases | 578 | 43,437 | (40,036-47,126) | 709 | 43,386 | (40,307-46,700) |
| Tubulointerstitial Disease | 377 | 41,976 | (37,946-46,435) | 515 | 50,673 | (46,480-55,244) |
| Other | 292 | 46,159 | (41,157-51,768) | 434 | 47,857 | (43,560-52,578) |
| **BMI Groups** |  |  |  |  |  |  |
| Not Obese | 1,903 | 36,395 | (34,796-38,067) | 3,352 | 40,666 | (39,312-42,066) |
| Obese | 822 | 40,368 | (37,700-43,224) | 1,512 | 47,043 | (44,730-49,475) |

### **Table S9.** 1-, 3- and 5-year cumulative incidence (CI) percentages of time from waitlisting to receiving a deceased donor transplant, by year eras (A) 2006-2010, (B) 2011-2015, (C) 2016-2019, and (D) 2020-2023.

| **Cumulative Incidence (%)** | (A) **Cumulative incidence of time from waitlisting to deceased donor transplant for year era 2006-2010** | | | |  |
| --- | --- | --- | --- | --- | --- |
|  | **Female** | | **Male** | |  |
|  | **CI (%)** | **95% CI** | **CI (%)** | **95% CI** | |
| **1-year** |  |  |  |  | |
| Received deceased donor transplant | 24.4 | (21.0-27.9) | 26.6 | (23.8-29.4) | |
| Died before transplant | 0.3 | (0.1-1.2) | 0.4 | (0.1-1.0) | |
| Received living donor transplant | 6.5 | (4.7-8.7) | 5.9 | (4.5-7.5) | |
| End of follow-up | NA |  | NA |  | |
| **3-years** |  |  |  |  | |
| Received deceased donor transplant | 48.0 | (43.9-52.0) | 53.3 | (50.0-56.4) | |
| Died before transplant | 1.4 | (0.7-2.6) | 1.7 | (1.0-2.7) | |
| Received living donor transplant | 10.1 | (7.8-12.7) | 10.2 | (8.4-12.2) | |
| End of follow-up | NA |  | NA |  | |
| **5-years** |  |  |  |  | |
| Received deceased donor transplant | 62.5 | (58.5-66.3) | 67.7 | (64.6-70.5) | |
| Died before transplant | 2.9 | (1.8-4.5) | 3.6 | (2.5-4.9) | |
| Received living donor transplant | 11.8 | (9.3-14.5) | 11.0 | (9.1-13.1) | |
| End of follow-up | NA |  | NA |  | |

| **Cumulative Incidence (%)** | (B) **Cumulative incidence of time from waitlisting to deceased donor transplant for year era 2011-2015** | | | |  |
| --- | --- | --- | --- | --- | --- |
|  | **Female** | | **Male** | |  |
|  | **CI (%)** | **95% CI** | **CI (%)** | **95% CI** | |
| **1-year** |  |  |  |  | |
| Received deceased donor transplant | 41.9 | (38.8-44.9) | 42.6 | (40.3-44.8) | |
| Died before transplant | 0.2 | (0.0-0.7) | 0.1 | (0.0-0.4) | |
| Received living donor transplant | 4.8 | (3.6-6.2) | 5.0 | (4.1-6.1) | |
| End of follow-up | NA |  | NA |  | |
| **3-years** |  |  |  |  | |
| Received deceased donor transplant | 67.5 | (64.6-70.3) | 69.6 | (67.5-71.7) | |
| Died before transplant | 1.4 | (0.8-2.2) | 1.8 | (1.2-2.4) | |
| Received living donor transplant | 7.0 | (5.5-8.6) | 7.4 | (6.3-8.7) | |
| End of follow-up | NA |  | NA |  | |
| **5-years** |  |  |  |  | |
| Received deceased donor transplant | 79.9 | (77.4-82.3) | 80.8 | (78.9-82.5) | |
| Died before transplant | 2.7 | (1.9-3.8) | 3.5 | (2.7-4.4) | |
| Received living donor transplant | 7.2 | (5.7-8.9) | 8.0 | (6.8-9.2) | |
| End of follow-up | NA |  | NA |  | |

| **Cumulative Incidence (%)** | (C) **Cumulative incidence of time from waitlisting to deceased donor transplant for year era 2016-2019** | | | |  |
| --- | --- | --- | --- | --- | --- |
|  | **Female** | | **Male** | |  |
|  | **CI (%)** | **95% CI** | **CI (%)** | **95% CI** | |
| **1-year** |  |  |  |  | |
| Received deceased donor transplant | 49.7 | (46.7-52.5) | 51.7 | (49.6-53.9) | |
| Died before transplant | 0.7 | (0.3-1.3) | 0.8 | (0.5-1.2) | |
| Received living donor transplant | 3.9 | (2.9-5.2) | 3.3 | (2.6-4.1) | |
| End of follow-up | NA |  | NA |  | |
| **3-years** |  |  |  |  | |
| Received deceased donor transplant | 70.5 | (67.8-73.1) | 71.6 | (69.6-73.5) | |
| Died before transplant | 2.8 | (2.0-3.9) | 3.1 | (2.4-3.9) | |
| Received living donor transplant | 5.7 | (4.4-7.1) | 5.7 | (4.7-6.7) | |
| End of follow-up | NA |  | NA |  | |
| **5-years** |  |  |  |  | |
| Received deceased donor transplant | 79.1 | (76.6-81.4) | 81.0 | (79.2-82.6) | |
| Died before transplant | 5.2 | (4.0-6.6) | 4.9 | (4.0-5.9) | |
| Received living donor transplant | 6.1 | (4.8-7.6) | 5.8 | (4.9-6.9) | |
| End of follow-up | 3.9 | (2.9-5.2) | 4.1 | (3.3-5.0) | |

| **Cumulative Incidence (%)** | (D) **Cumulative incidence of time from waitlisting to deceased donor transplant for year era 2020-2023** | | | |  |
| --- | --- | --- | --- | --- | --- |
|  | **Female** | | **Male** | |  |
|  | **CI (%)** | **95% CI** | **CI (%)** | **95% CI** | |
| **1-year** |  |  |  |  | |
| Received deceased donor transplant | 33.6 | (31.0-36.2) | 34.0 | (32.0-36.0) | |
| Died before transplant | 0.7 | (0.4-1.3) | 0.8 | (0.5-1.2) | |
| Received living donor transplant | 4.5 | (3.5-5.8) | 4.1 | (3.3-5.0) | |
| End of follow-up | 20.6 | (18.4-22.8) | 18.3 | (16.7-20.0) | |
| **3-years** |  |  |  |  | |
| Received deceased donor transplant | 45.2 | (42.4-48.0) | 45.6 | (43.4-47.7) | |
| Died before transplant | 1.5 | (1.0-2.3) | 2.7 | (2.1-3.5) | |
| Received living donor transplant | 6.5 | (5.2-7.9) | 5.9 | (4.9-6.9) | |
| End of follow-up | 40.6 | (37.9-43.3) | 39.9 | (37.8-42.0) | |
| **5-years** |  |  |  |  | |
| Received deceased donor transplant | 46.4 | (43.6-49.2) | 47.0 | (44.9-49.1) | |
| Died before transplant | 1.7 | (1.1-2.5) | 2.8 | (2.2-3.6) | |
| Received living donor transplant | 6.5 | (5.2-7.9) | 5.9 | (4.9-6.9) | |
| End of follow-up | 45.5 | (42.7-48.2) | 44.3 | (42.2-46.5) | |

### **Table S10.** Rates per 100,000 person-years for dying before receiving a kidney transplant.

| **Rates per 100,000 Person-Years** | (A) **Waitlisting to Death Before Transplant** | | | | | |
| --- | --- | --- | --- | --- | --- | --- |
|  | **Female** | | | **Male** | | |
|  | **Count** | **Rate** | **95% CI** | **Count** | **Rate** | **95% CI** |
| **Sex** | 221 | 3,006 | (2,634-3,429) | 380 | 3,281 | (2,968-3,628) |
| **Age at dialysis initiation (years)** |  |  |  |  |  |  |
| <18 | 1 | 996 | (140-7,068) | 0 | 0 | 0 |
| 18-45 | 47 | 1,869 | (1,405-2,488) | 55 | 1,594 | (1,224-2,076) |
| 46-65 | 136 | 3,310 | (2,798-3,916) | 240 | 3,557 | (3,134-4,036) |
| >65 | 37 | 5,875 | (4,257-8,109) | 85 | 6,982 | (5,645-8,636) |
| **Ethnicity** |  |  |  |  |  |  |
| Australian, New Zealander/European | 133 | 3,111 | (2,625-3,688) | 246 | 3,486 | (3,077-3,950) |
| Aboriginal & Torres Strait Islander | 24 | 5,033 | (3,373-7,509) | 25 | 3,869 | (2,615-5,726) |
| Māori & Pacific Peoples | 18 | 4,347 | (2,739-6,899) | 19 | 3,915 | (2,497-6,138) |
| Asian | 37 | 2,267 | (1,643-3,129) | 68 | 3,005 | (2,369-3,811) |
| Other | 9 | 1,620 | (843-3,114) | 22 | 1,947 | (1,282-2,956) |
| **Socio-Economic Position** |  |  |  |  |  |  |
| Lowest 20% (Most disadvantaged) | 62 | 3,565 | (2,780-4,573) | 91 | 3,568 | (2,905-4,382) |
| Highest 80% (Least disadvantaged) | 158 | 2,849 | (2,438-3,330) | 286 | 3,197 | (2,847-3,589) |
| **Comorbidity Burden** |  |  |  |  |  |  |
| 0 | 64 | 1,514 | (1,185-1,934) | 84 | 1,442 | (1,165-1,786) |
| 1 | 83 | 4,264 | (3,439-5,287) | 121 | 3,781 | (3,164-4,518) |
| 2 | 44 | 5,460 | (4,063-7,336) | 93 | 6,101 | (4,979-7,476) |
| ≥3 | 30 | 8,045 | (5,625-11,506) | 82 | 7,946 | (6,400-9,867) |
| **Cause of Kidney Failure** |  |  |  |  |  |  |
| Diabetic Kidney Disease | 95 | 6,650 | (5,439-8,132) | 193 | 6,867 | (5,964-7,908) |
| Hypertension/Renal Vascular Disease | 16 | 2,761 | (1,692-4,507) | 43 | 3,324 | (2,465-4,482) |
| GN | 43 | 1,731 | (1,284-2,334) | 75 | 1,914 | (1,526-2,400) |
| Familial/Hereditary Kidney Diseases | 20 | 1,503 | (970-2,330) | 25 | 1,530 | (1,034-2,264) |
| Tubulointerstitial Disease | 29 | 3,229 | (2,244-4,647) | 17 | 1,673 | (1,040-2,691) |
| Other | 18 | 2,845 | (1,793-4,516) | 27 | 2,977 | (2,042-4,341) |
| **BMI Groups** |  |  |  |  |  |  |
| Not Obese | 150 | 2,869 | (2,445-3,367) | 257 | 3,118 | (2,759-3,523) |
| Obese | 67 | 3,290 | (2,590-4,181) | 117 | 3,640 | (3,037-4,363) |
